# Identification of RNA N6-methyladenosine regulation in epilepsy: Significance of the cell death mode, glycometabolism, and drug reactivity

**DOI:** 10.3389/fgene.2022.1042543

**Published:** 2022-11-17

**Authors:** Xuchen Liu, Qingyuan Sun, Zexin Cao, Wenyu Liu, Hengrui Zhang, Zhiwei Xue, Jiangli Zhao, Yifei Feng, Feihu Zhao, Jiwei Wang, Xinyu Wang

**Affiliations:** ^1^ Department of Neurosurgery, Qilu Hospital, Cheeloo College of Medicine and Institute of Brain and Brain-Inspired Science, Shandong University, Jinan, China; ^2^ Jinan Microecological Biomedicine Shandong Laboratory and Shandong Key Laboratory of Brain Function Remodeling, Jinan, China; ^3^ School of Medicine, Cheeloo College of Medicine, Shandong University, Jinan, China

**Keywords:** epilepsy, m6A RNA methylation regulators, glycometabolism, cuproptosis, ferroptosis

## Abstract

Epilepsy, a functional disease caused by abnormal discharge of neurons, has attracted the attention of neurologists due to its complex characteristics. N6-methyladenosine (m6A) is a reversible mRNA modification that plays essential role in various biological processes. Nevertheless, no previous study has systematically evaluated the role of m6A regulators in epilepsy. Here, using gene expression screening in the Gene Expression Omnibus GSE143272, we identified seven significant m6A regulator genes in epileptic and non-epileptic patients. The random forest (RF) model was applied to the screening, and seven m6A regulators (HNRNPC, WATP, RBM15, YTHDC1, YTHDC2, CBLL1, and RBMX) were selected as the candidate genes for predicting the risk of epilepsy. A nomogram model was then established based on the seven-candidate m6A regulators. Decision curve analysis preliminarily showed that patients with epilepsy could benefit from the nomogram model. The consensus clustering method was performed to divide patients with epilepsy into two m6A patterns (clusterA and clusterB) based on the selected significant m6A regulators. Principal component analysis algorithms were constructed to calculate the m6A score for each sample to quantify the m6A patterns. Patients in clusterB had higher m6A scores than those in clusterA. Furthermore, the patients in each cluster had unique immune cell components and different cell death patterns. Meanwhile, based on the M6A classification, a correlation between epilepsy and glucose metabolism was laterally verified. In conclusion, the m6A regulation pattern plays a vital role in the pathogenesis of epilepsy. The research on m6A regulatory factors will play a key role in guiding the immune-related treatment, drug selection, and identification of metabolism conditions and mechanisms of epilepsy in the future.

## Introduction

Epilepsy is a common brain condition characterized by the recurrence of unprovoked seizures, with over 70 million patients affected worldwide (Thijs et al., 2019). The clinical manifestations of epilepsy are complex and diverse, including loss of consciousness, muscle rigidity, limb clonus, the disappearance of muscle tension and other symptoms, which negatively impacts the patients’ quality of life and imparts a significant economic burden (Covanis et al., 2015; Saxena and Li, 2017). Recent research has shown that epilepsy has a strong genetic predisposition and is related to multiple groups of explicit and recessive genes, which are often shared with other functional neurological diseases such as paroxysmal dyskinesia (Reid et al., 2009; Gardiner et al., 2012; Koeleman, 2018). Treatment for epilepsy is complicated. Recently, psychotherapy and diet therapy have been put forward (Elia et al., 2017; Nigro, 2019), but drug treatment is still the focus of epilepsy symptom control (Mirza et al., 2021). Traditional epilepsy drugs target ion channels and neurotransmitter receptors (Hahn and Neubauer, 2009; Zaccara and Perucca, 2014), and there is currently insufficient knowledge to design stable and effective antiepileptic drugs by using specific genes and their products as intervention sites.

N6-methyladenosine (m6A) is the most prevalent internal modification of eukaryotic mRNA. Recent research elucidated the roles of RNA modifications in modulating gene expression, including cellular self-renewal, differentiation, invasion, and apoptosis (Liu et al., 2020; Liu and Su, 2021; Zhu et al., 2021). M6A affected the stability and translation of the modified transcripts and provided a mechanism to coordinate the transcripts regulation during cellular state maintenance and transition (Wei and He, 2021). Previous studies have confirmed that the m6A modification is inversely associated with mRNA stability and gene expression, which are also useful in cancer research (Wang et al., 2014; He et al., 2019). As an epigenetic modification that requires multiple regulatory proteins encoded by genes called writers, erasers, and readers (Yang et al., 2018; Shi et al., 2019), many non-tumor studies have also begun considering the relationship between m6A and related diseases such as Parkinson’s disease (Qin et al., 2020). Numerous studies have focused on de-methylated transferase (FTO, ALKBH5), which can maintain a dynamic balance between the methylation and de-methylation of mRNA (Rowles et al., 2012). However, there is still a lack of research on the roles of m6A regulators in epilepsy.

Gene Expression Omnibus (GEO) GSE143272 dataset has been widely cited in epilepsy studies (Luo et al., 2021). In this study, GSE143272 was analyzed to evaluate the functions of m6A regulators in the diagnosis and subtype classification of patients with epilepsy. We established a gene model for predicting the correlation between the prediction of epilepsy susceptibility and drug treatment based on the model with seven candidate m6A regulators. As a result, we found that the model could provide good clinical prediction value and be used to analyze the effects of common drugs, such as carbamazepine, phenytoin, and valproic acid, on m6A gene expression. Additionally, we revealed two distinct m6A patterns that were highly consistent with the expression of autophagy-, ferroptosis-, and cuproptosis-related genes, suggesting that m6A patterns may be used to distinguish patients with epilepsy by the activation of different induced cell death pathways that are implicated in the pathogenesis of cell death. Finally, the gene expression results were verified by clinical samples from epilepsy patients.

## Materials and methods

### Data acquisition

Gene expression information from 91 patients with epilepsy and 51 healthy controls were obtained from the GEO database (https://www.ncbi.nlm.nih.gov/geo/) (Barrett et al., 2013), all of whom were acquired from the GSE143272 dataset. By identifying significant m6A regulators between patients with epilepsy and healthy controls, 17 m6A regulators were extracted from the dataset, comprising six writers (METTL3, METTL14, WTAP, RBM15, RBM15B, and CBLL1), ten readers (YTHDC1, YTHDC2, YTHDF1, YTHDF2, YTHDF3, HNRNPC, LRPPRC, HNRNPA2B1, RBMX, and ELAVL1), and one eraser (ALKBH5). Furthermore, clinical information was also obtained, including age, sex, subtype, and patients’ response to different antiepileptic drugs.

### Construction of the support vector machine model and random forest model.

A random forest (RF) and support vector machine (SVM) were constructed to predict the occurrence of epilepsy. To evaluate the two models above, the “Reverse cumulative distribution of residual,” “Boxplots of residual,” and receiver operating characteristic (ROC) curve were plotted. RF is a constituent supervised learning classifier containing multiple decision trees, which provide a widely used QSAR method with high prediction accuracy. We used the “Randomforest” package in R statistical software (The R Foundation, Vienna, Austria) to establish an RF model and selected candidate m6A regulators among the seven differentially expressed m6A regulators to predict the occurrence of epilepsy. In this research, ntrees and mtry were set at 55 and 3, respectively, and through 10-fold cross-validation, seven appropriate important m6A regulators were selected after analyzing their importance (Qin et al., 2019).

### Construction of the nomogram model

To predict the prevalence of patients with epilepsy, a nomogram model based on the selected candidate m6A regulators was constructed using the “rms” package in R, and the calibration curve was formed to evaluate the consistency between our predicted values and reality. Decision curve analysis (DCA) and clinical impact were also performed to judge whether the decisions based on the model benefited patients with epilepsy (Iasonos et al., 2008).

### Feature analysis of molecular subtypes based on the significant m6A regulators

To identify distinct m6A patterns based on the significant m6A regulators, we performed the consensus clustering method using the “ConsensusClusterPlus” package in R (Wilkerson and Hayes, 2010). The consensus clustering used above was an algorithm with a function to identify each number and its subgroup number, which was then verified using clustering rationality based on resampling.

### Identification of differentially expressed genes between distinct m6A patterns

Differentially expressed genes (DEGs) between distinct m6A patterns were analyzed and screened using the “limma” package with *p* < 0.05 and |logFC| > 0.35 as the screening criterion (the cut-off criteria for statistical significance were a log fold change (FC) of >0.35 and a *p*-value of <0.05) (Ritchie et al., 2015).

### Functional and pathway enrichment analysis

Gene Ontology (GO) functional enrichment was applied to explore the enrichment pathways and functional annotation with the aid of the “clusterProfiler” package (Ashburner et al., 2000). Furthermore, Gene Set Enrichment Analysis (GSEA) was conducted to analyze the differently expressed pathways in two distinct m6A patterns using GSEA software (v4.2.3). The enrichment scores (ES) were calculated based on weighted Kolmogorov–Smirnov-like statistics, whose magnitude can imply the relationship between a get set and the group. The higher ES of the gene set, the higher the possibility that it was considered to be enriched in a particular group (Subramanian et al., 2005). Furthermore, the pathway maps were drawn using pathview (https://pathview.uncc.edu/), a server for pathway-based visualization and data integration (Luo et al., 2017).

### Estimation of the m6A gene signature

Principal component analysis (PCA) was used to distinguish the m6A patterns. The m6A score was calculated according to the formula: m6A score = PC1_i_, where PC1 is principal component 1, representing the DEGs. The M6A scores for each sample were calculated using PCA algorithms (Ringnér, 2008; Chong et al., 2021).

### Estimation of immune cell infiltration

Single-sample gene set enrichment analysis (ssGSEA) was conducted to analyze and calculate the type and abundance of 23 types of immune cells that are commonly infiltrated in patients with epilepsy. The gene expression levels in these samples were sequenced to obtain their rank, and their expression levels were then summed from the input dataset to obtain the abundance of immune cells in each sample (Manford, 2017).

### RNA extraction and qRT-PCR

TRIzol was used to extract the total cell RNA according to the manufacturer’s protocol. Reverse transcription was performed using High Capacity cDNA Reverse Transcription Kits (Applied Bio-systems) according to the manufacturer’s protocols. The cDNA was subjected to real-time PCR using the quantitative PCR System Mx-3000P (Stratagene). The PCR primer sequences can be found in Supplementary Table S1.

### Statistical analysis

Linear regression analyses were used to predict the relationship between erasers and writers, and the correlation between the 17 extracted m6A genes. Differences between the groups were detected by Kruskal–Wallis tests. All parametric analyses were built on a two-tailed test, with a statistic significance set at *p* < 0.05. All statistical analyses were performed using R version 4.1.2.

## Results

### The difference in the expression of M6A regulators in epilepsy

**FIGURE 1:**
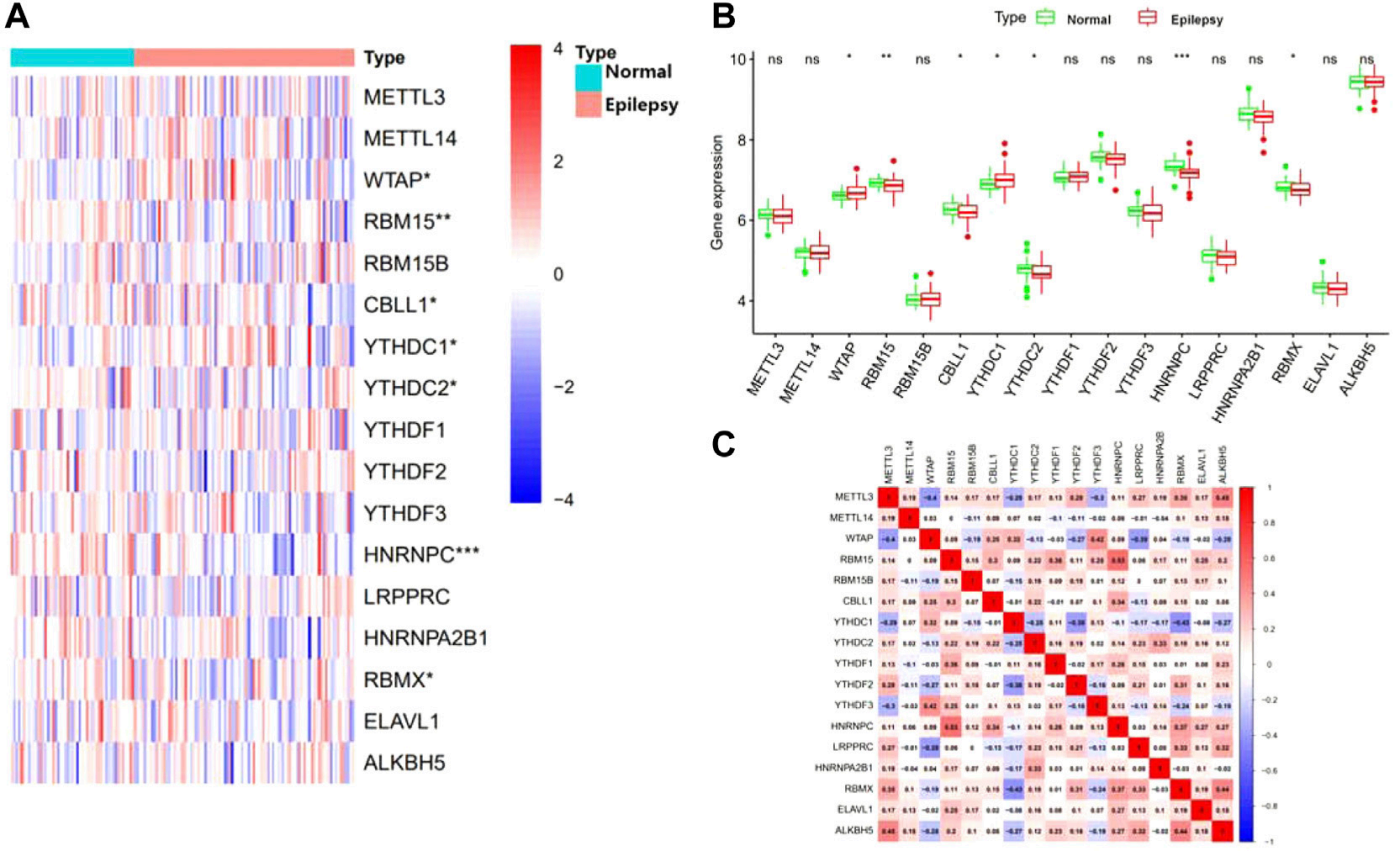
Landscape of the 17 RNA N6-methyladenosine (m6A) regulators in epilepsy. **(A)** Expression heat map of the m6A regulators in patients with epilepsy and healthy groups. **(B)** Differential expression histogram of 17 m6A regulators between non-epilepsy and patients with epilepsy. **(C)** Spearman correlation analysis of 17 m6A regulators. *p < 0.05, **p < 0.01, and ***p < 0.001.

Seventeen m6A regulators were analyzed with the differential expression levels between patients with epilepsy and normal groups using the “limma” package. Seven significant m6A regulators, consisting of three writers (WTAP, RBM15, and CBLL1) and four readers (YTHDC1, YTHDC2, HNRNPC, and RBMX) were screened and selected. The boxplot and heatmap intuitively show the expression of these differential genes (*p*-value < 0.05) (Figures 1A,B). These findings indicated that WTAP and YTHDC1 were overexpressed in patients with epilepsy, while RBM15, CBLL1, YTHDC2, HNRNPC, and RBMX showed decreased expression in patients with epilepsy compared to that in the healthy group.

### Correlation analysis between M6A-Related genes in epilepsy

**FIGURE 2:**
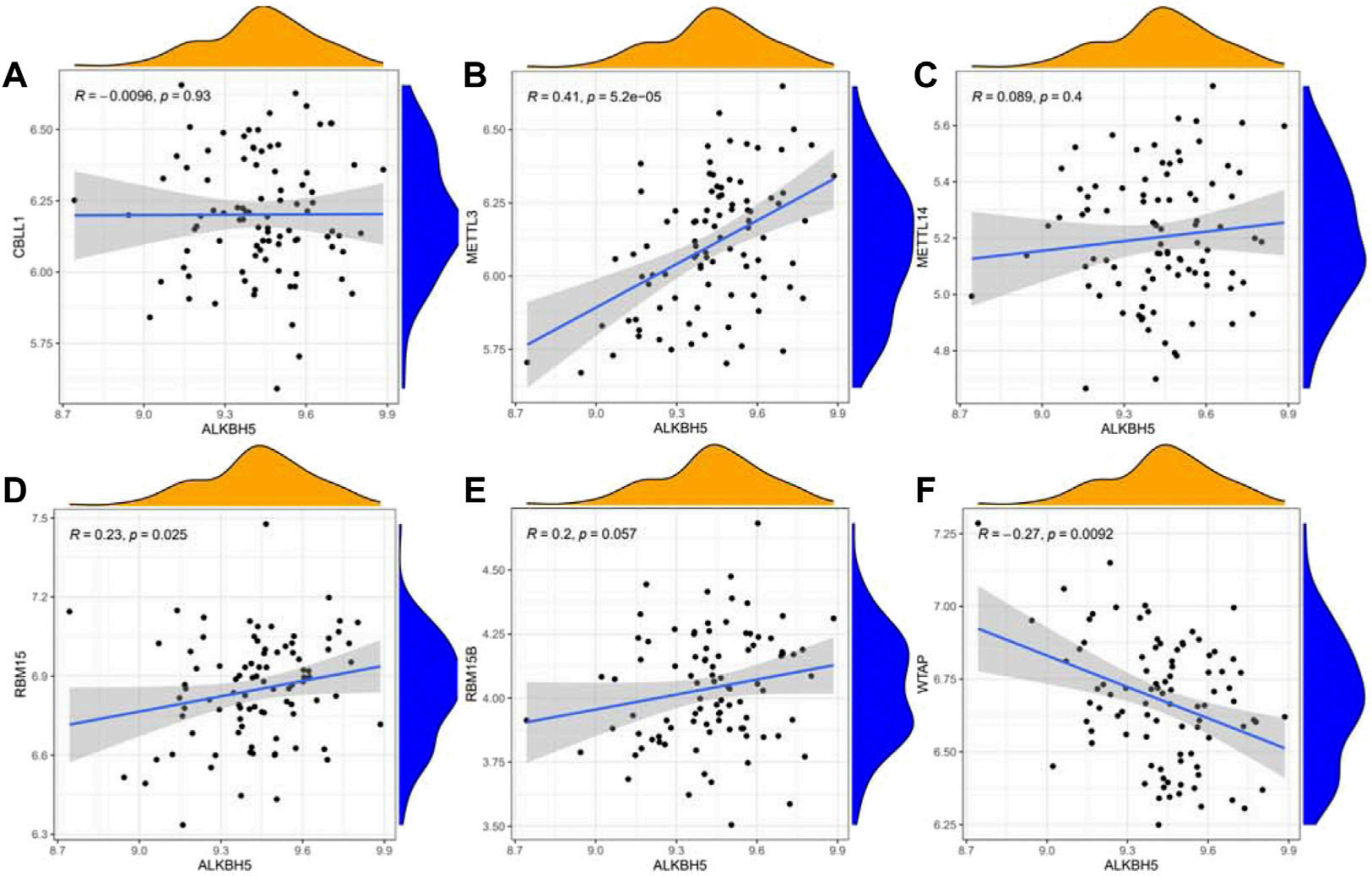
Linear regression analyses shows the correlation between one eraser gene and six writer genes in epilepsy **(A–F)**. Eraser gene: ALKBH5. Writer genes: CBLL1, METTL3, METTL14, RBM15, RBM15B, and WTAP.

We next performed a correlation matrix to analyze whether there was a correlation between m6A-related gene expression levels in epilepsy samples (Figure 1C). We found a positive correlation between the expression of HNRNPC, RBMX, and RBM15 in patients with epilepsy. The expression of YTHDC1 was negatively correlated with RBMX and YTHDF2, with the same result observed between WTAP, LRPPRC, and METTL3. We also analyzed the relationship between the readers and the eraser (ALKBH5) (Figures 2A–F). The results showed that ALKBH5 is positively correlated with RBM15 and METTL3, but has an opposite relationship with WTAP.

### Construction of the RF model and SVM model

**FIGURE 3:**
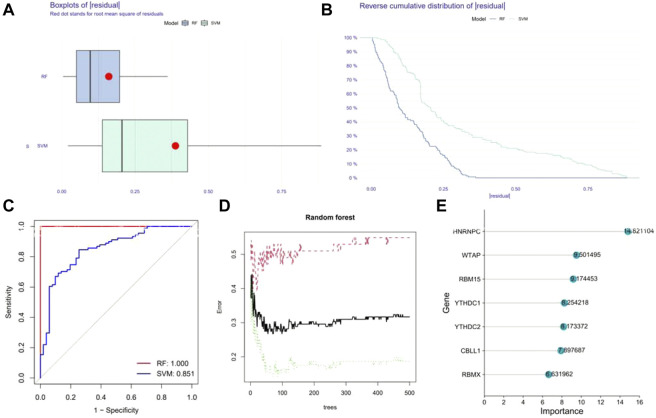
Construction of Random forest (RF) model. **(A)** The residual distribution of the RF and SVM model. **(B)** Reverse cumulative distribution of the residual of the RF and SVM model. **(C)** Receiver Operating Characteristic (ROC) curves verifying the accuracy of the RF and SVM model. **(D)** Correlation between the error and number of trees of non-epilepsy individuals, patients with epilepsy, and both. **(E)** The score of the importance of seven significant m6A regulators using the RF model (importance >2).

Reverse cumulative distribution of the residual and Boxplots of the residual were established to estimate the RF and SVM models to select candidate m6A regulators from the 17 m6A regulators to predict the occurrence of epilepsy. The results both showed that the RF model had minimal residuals (Figures 3A,B), while the ROC curve plotted to evaluate the model also indicated that the RF model had higher accuracy than the SVM model with the AUC value of the ROC curve (Figure 3C). With the smaller residuals, which are considered better for evaluation, the RF model was selected to predict the occurrence of epilepsy, with the ntrees and mtry set at 55 and 3, respectively (Figure 3D). We next rated the score of importance of these seven differently expressed m6A regulators (HNRNPC, WATP, RBM15, YTHDC1, YTHDC2, CBLL1, RBMX) and selected them as the candidate genes (Figure 3E).

### Construction of the nomogram model

**FIGURE 4:**
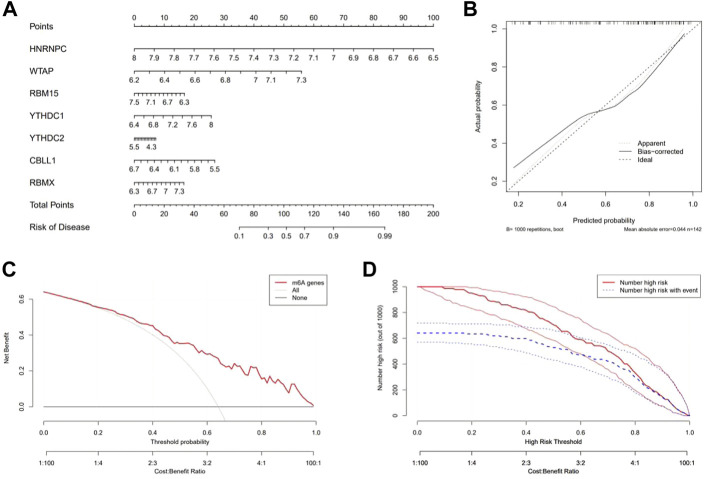
Construction and benefit prediction of the nomogram model. **(A)** Nomogram plotted to predict the occurrence of epilepsy based on seven significant m6A regulators. **(B)** Calibration curve of the nomogram showing the consistency between the predicted and true occurrence of epilepsy. **(C)** Analysis of whether the nomogram model is beneficial to patients with epilepsy. **(D)** Clinical impact curve to show the clinical impact of the nomogram model.

By using the “rms” package, a nomogram model based on the seven candidate m6A regulators was constructed to predict the prevalence of epilepsy patients (Figure 4A). Calibration curves revealed that the predictivity of the nomogram model was accurate (Figure 4B). The red line in the DCA curve remained above the gray and black lines from 0 to 1, indicating that decisions based on the nomogram model may benefit patients with epilepsy (Figure 4C). The clinical impact curve revealed a remarkable predictive power of the nomogram model (Figure 4D).

### Two distinct m6A patterns identified by significant m6A regulators

**FIGURE 5:**
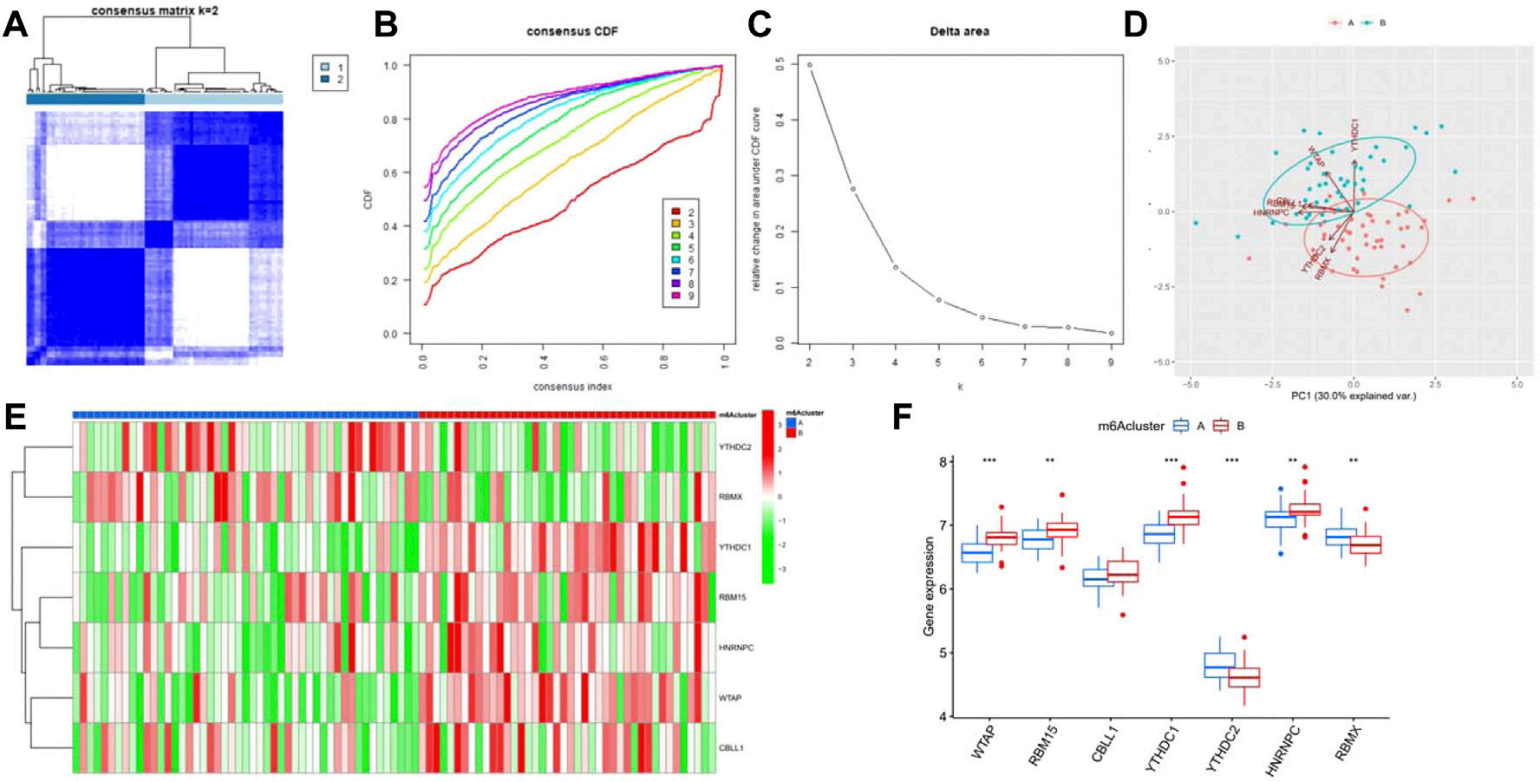
Consensus clustering of the significant RNA N6-methyladenosine (m6A) regulators in epilepsy. **(A)** Consensus score matrix of patients with epilepsy for k = 2. **(B)** Correlation between cumulative distribution functions. **(C)** Relative change in the area under the CDF curve for k = 2–9. **(D)** Principal component analysis for the expression profiles of the significant m6A regulators between the two m6A patterns. **(E)** Heatmap of seven significant m6A regulators between m6Acluster A and m6Acluster **(B) (F)** Differential expression of the seven significant m6A regulators between m6Acluster A and m6AclusterB. *p < 0.05, **p < 0.01, and ***p < 0.001.

Based on the seven significant m6A regulators and the “ConsensusClusterPlus” package, the patients with epilepsy were clustered into different subgroups using the consensus clustering method. It was shown that when K = 2, the consensus matrix has the cleanest distinguishing ability, and the interference between subgroups is minimal (Figures 5A–C), with 49 cases in clusterA and 42 cases in clusterB. PCA indicated that the m6A regulators could distinguish the patients into two m6A patterns distinctly (Figure 5D). The heatmap and boxplot revealed the differential expression levels of the seven significant m6A regulators between the two clusters, showing that YTHDC2 and RBMX displayed higher expression levels in clusterA than in clusterB. WTAP, RBM15, YTHDC1, and HNRNPC showed lower expression levels in clusterA, while CBLL1 showed no significant differences between the two clusters (Figures 5E,F). A total of 87 m6A-related DEGs were selected between the two m6A patterns.

### Enrichment analysis of screening differential genes between patterns

**FIGURE 6:**
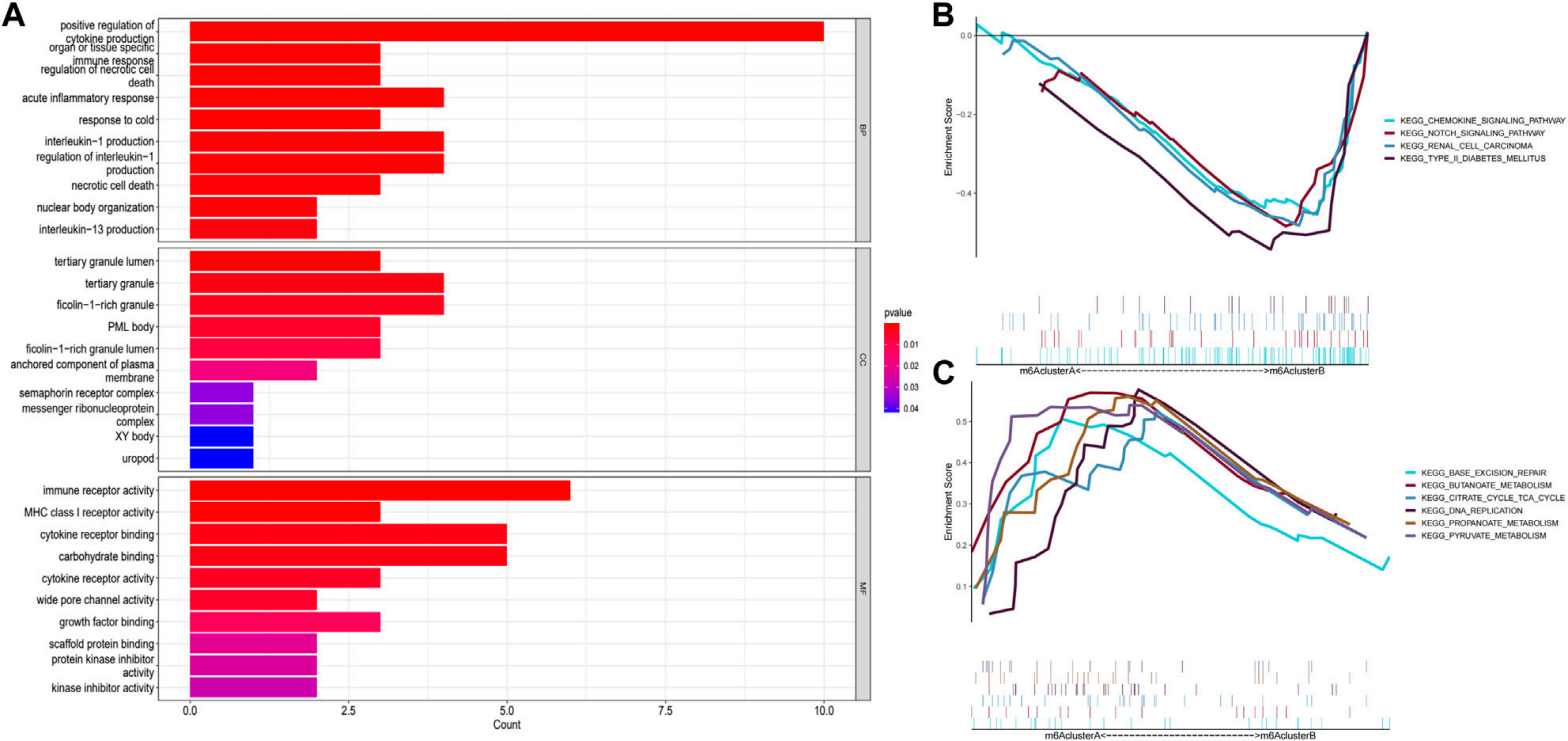
Enrichment analysis of gene expression in samples. **(A)** Graph showing the Gene Ontology (GO) analysis of the related pathways underlying the effect of the 87 m6A-related differentially expressed genes (DEGs) in patients with epilepsy. **(B,C)** Gene Set Enrichment Analysis (GSEA) to predict the pathway of DEGs affecting the occurrence and progression of epilepsy (*p* < 0.05).

GO enrichment analysis and GSEA were performed to explore the related pathways involved in m6A-related DEGs in patients with epilepsy. The results showed that the mainly centralized pathways were “positive regulation of cytokine production” in Biological Process (BP), “tertiary granule lumen” in Cellular Component (CC), and “immune receptor activity” in Molecular Function (MF) (Figure 6A). Moreover, the results for GSEA suggested that several pathways were dynamically enriched in the m6A cluster A compared to those in the m6A cluster B, including base excision repair, citrate cycle, TCA cycle, and intestinal immune network for IGA production. (Figure 6B), while axon guidance, chemokine signaling pathway, cytokine-cytokine receptor interaction, notch signaling pathway, renal cell carcinoma, and type II diabetes mellitus were enriched in the m6A cluster B (Figure 6C). The enrichment results of GO and GSEA suggested that these differential genes are closely related to immune function, cell death, and glycometabolism.

### Identification of genotyping of subtype signatures for the m6A gene by the consensus clustering method

**FIGURE 7:**
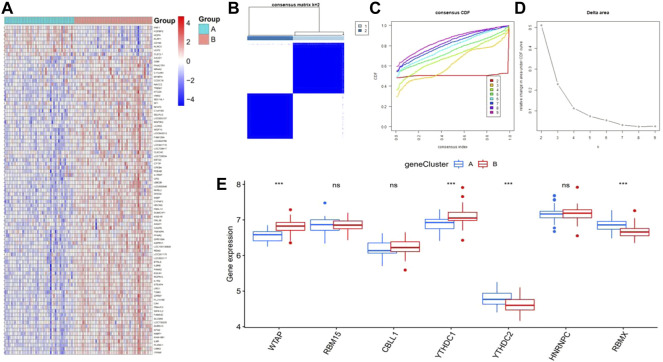
Consensus clustering of the 87 RNA N6-methyladenosine (m6A)-related differentially expressed genes (DEGs) in epilepsy, ILogFCI>0.35, *p*-value<0.05. **(A)** Expression heatmap of the 87 m6A-related DEGs between gene clusterA and gene clusterB. **(B–D)** Consensus matrix of k = 2, as well as the CDF curve and relative change in the area under the CDF curve for k = 2–9 **(E)** Differential expression of seven significant m6A regulators between gene cluster A and gene cluster **(B)** *p < 0.05, **p < 0.01, and ***p < 0.001.

We divided the patients with epilepsy into different genomic subtypes based on the 87 m6A-related DEGs into gene clusterA and gene clusterB and visualized their expression between these groups in the heatmap (Figure 7A). This grouping method was consistent with the sectionalization of m6A patterns (Figures 7B–D). A boxplot was also drawn to show the differential expression of the seven significant m6A regulators between the two gene clusters, which revealed that the expression of YTHDC2 and RBMX were higher in gene clusterA, while WTAP and YTHDC1 were expressed at lower levels (Figure 7E).

### Calculation of the abundance of immune cells and the difference between m6A and gene clusters

**FIGURE 8:**
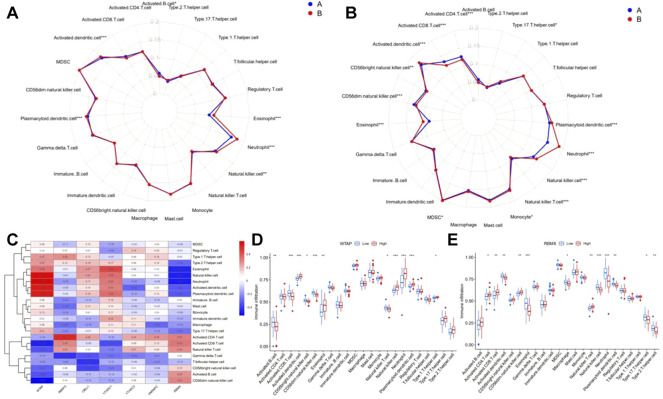
Immune characteristics between m6A and gene clusters. **(A,B)** Radar plot showing the 23 types of immune cells differing between m6A and gene clusters. **(C)** The correlation between the abundance of immune cells and seven significant m6A regulators. **(D–E)** Immune infiltration of the differential expression group of RBMX and WTAP. *p < 0.01, **p < 0.001, ***p < 0.0001.

Next, ssGSEA was applied to calculate the abundance of immune cells in patients with epilepsy. The results showed that the number of immune cells was different between m6A clusters (Figure 8A) and gene clusters (Figure 8B). According to the expression of the seven significant m6A regulators and the abundance of immune cells, the correlation between them was analyzed and displayed in Figure 8C, which revealed that WTAP and RBMX were distinctly correlated with numerous immune cells. We also found that both genes tended to show an antagonistic relationship, which is shown more clearly in the boxplots (Figures 8D,E).

### Differences in cuproptosis-, pyroptosis-, and ferroptosis-related genes between m6A clusters and gene clusters

**FIGURE 9:**
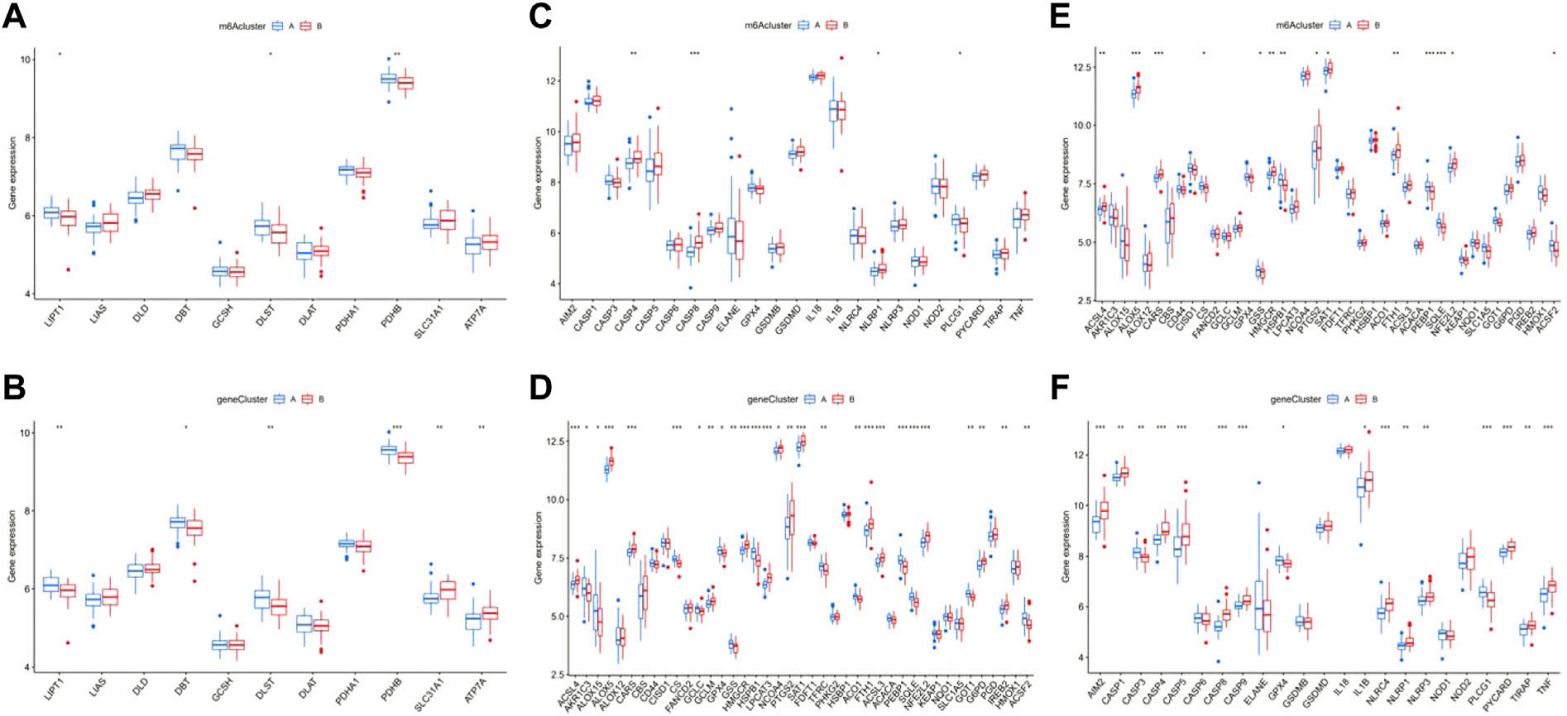
Correlation between the expression of cuproptosis, pyroptosis, and ferroptosis in different m6A clusters and gene clusters in epilepsy patients. The difference of expression of cuproptosis in different m6A clusters **(A)** and gene clusters **(B)**. The same difference analysis of pyroptosis **(C,D)** and ferroptosis **(E)**, **(F)** *p < 0.01, **p < 0.001, ***p < 0.0001.

The results of enrichment analysis implied a difference in cell death regulation between m6A clusters, and consequently, a more detailed level of verification was actualized. The results showed that multiple cuproptosis-related genes had distinct expression between m6A clusters and gene clusters, including LIPT1, DLST, and PDHB (Figures 9A,B). Following the same pathway, CASP4, CASP8, NLRP1, and PLCG1, included in the pyroptosis-related genes, were also differentially expressed (Figures 9C,D). Additionally, ACSL4, ALOX5, CARS, CS, GSS, HMGCR, HSPB1, PTGS2, SAT1, FTH1, PEBP1, SQLE, NFE2L2, and ACSF2 showed significant different expressions between m6A and gene clusters as ferroptosis genes (Figures 9E,F).

### Metabolic correlation analysis in m6A and gene clusters

**FIGURE 10:**
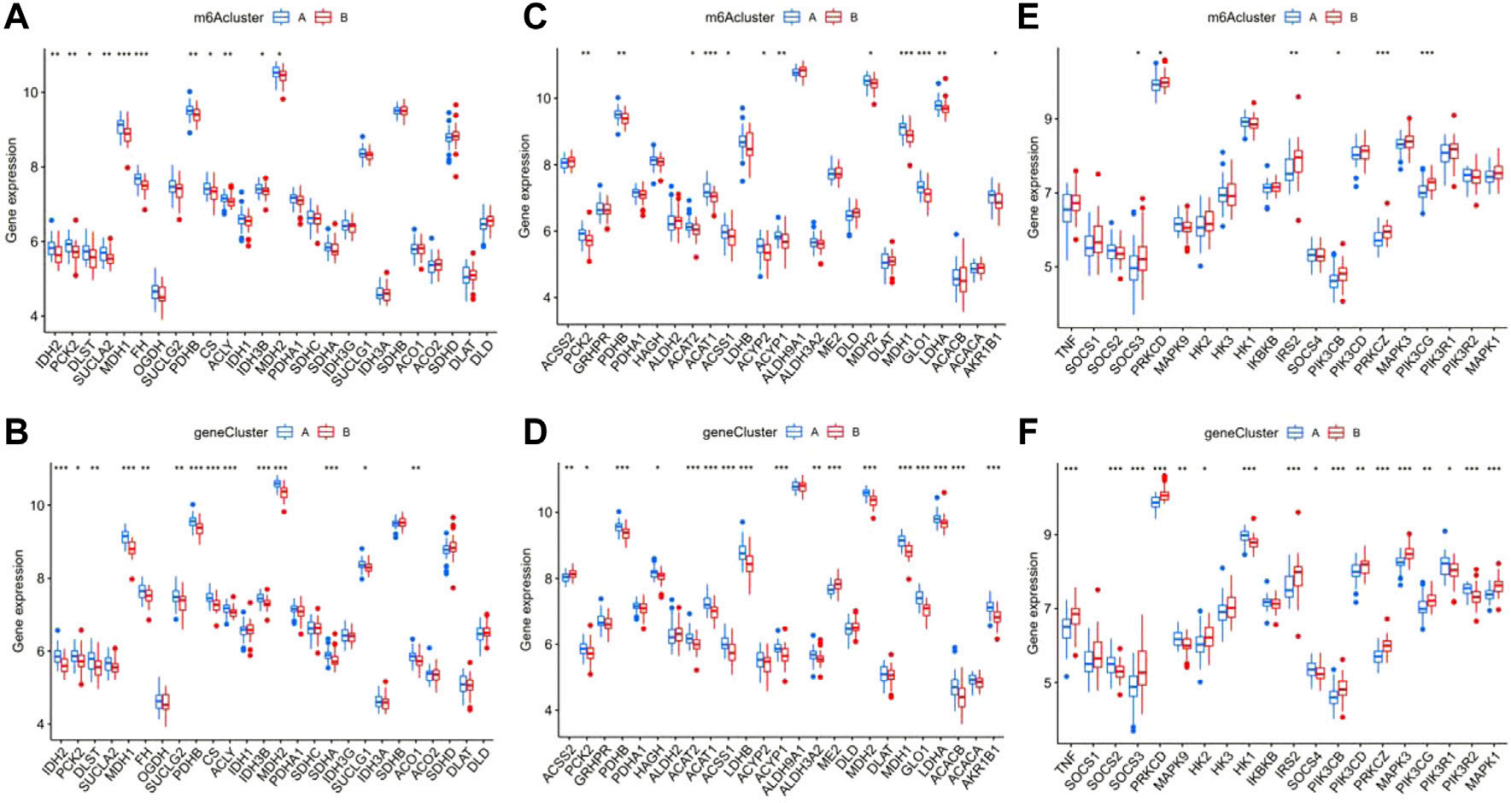
Analysis of the correlation between the different m6A and gene clusters in patients with epilepsy with the tricarboxylic acid cycle (TAO), pyruvate metabolism, and type II diabetes. The difference in expression of tac-related genes in m6A clusters **(A)** and gene clusters **(B)**, pyruvate metabolism **(C,D)**, and type II diabetes **(E,F)**.*p < 0.01, **p < 0.001, ***p < 0.0001.

We confirmed that tricarboxylic acid cycle (TAC)-, pyruvate metabolism-, and type II diabetes-related genes were significantly differentially expressed in patients with epilepsy and the healthy group, and preliminarily verified that there was a certain statistically significant correlation with the prognosis of patients. Based on this, we analyzed the correlation between TAC-related genes and m6A and gene clusters and found that many vital genes in TAC, such as PCK2, PDHB, ACAT, ACAT, ACSS1, MDH1, G1O1, LDHA, and AKRIB1, had significant differences between both m6A and gene clusters (Figures 10A,B). Using the same analysis model, we also found prominent differences between clusters in pyruvate metabolism and type II diabetes-related genes (Figures 10C–F). We further explored the differences in the TAC and type II diabetes mellitus pathways in clusters. The results indicated that the pathway of insulin resistance was activated in m6A cluster B Supplementary Figure S1A, while aerobic oxidation was inhibited in m6A cluster B in Supplementary Figure S1B, showing that the m6A cluster B was more likely to be associated with type II diabetes mellitus than the m6A cluster A.

### Correlation analysis between clinical characteristics and m6A score

**FIGURE 11:**
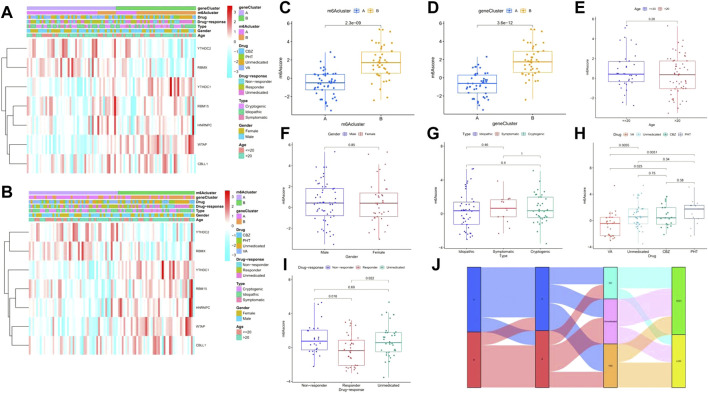
Relationship between the signature risk scores and clinical factors. **(A–B)** Heatmaps for the expression of 7 significant m6A regulators in m6A clusters and gene clusters with clinical features (age, gender, subtype, drug-response, and drug type). **(C–I)** The comparison of m6A score between or among different teams. **(J)** Sankey diagram to show the relation of m6A patterns, m6A-related DEGs patterns, Drug-response, and m6A score *p < 0.01, **p < 0.001, ***p < 0.0001.

In this study, PCA algorithms were conducted to calculate the m6A score for each sample as m6A patterns. Through the extraction of clinical data from the GEO dataset (Supplementary Table S1), we drew a clinical correlation heatmap related to the m6A score by displaying the expression of seven significant m6A regulators and analyzed its relationship with the m6A cluster, gene cluster, age, sex, subtype, drug, and drug response (Figures 11A,B). The results confirmed that the m6A score between the two distinct m6A patterns or m6A gene patterns was higher in clusterB or gene cluster B than that in clusterA or gene clusterA (Figures 11C,D). Moreover, the m6A scores were not statistically significant under the influence of age, sex, and subtype (Figures 11E–G). Notably, a difference was also found between patients who took valproic acid (VA) and other patients, including those who took carbamazepine (CBZ), phenytoin (PHT), and no drug treatment; however, the latter three groups of patients showed no significant differences in terms of m6A score (Figure 11H). Furthermore, in the samples with high pharmacodynamic response, the m6A score was significantly lower than that of the group without drugs or that with a poor drug response, which preliminarily proves that the m6A score can be used to predict the future pharmacodynamic response of patients with epilepsy (Figure 11I). Finally, a Sankey diagram was drawn to confirm the relationship between m6A patterns, m6A gene patterns, drug response, and the patients’ m6A scores (Figure 11J).

### Validation of m6A-Related genes by real-time PCR

**FIGURE 12:**
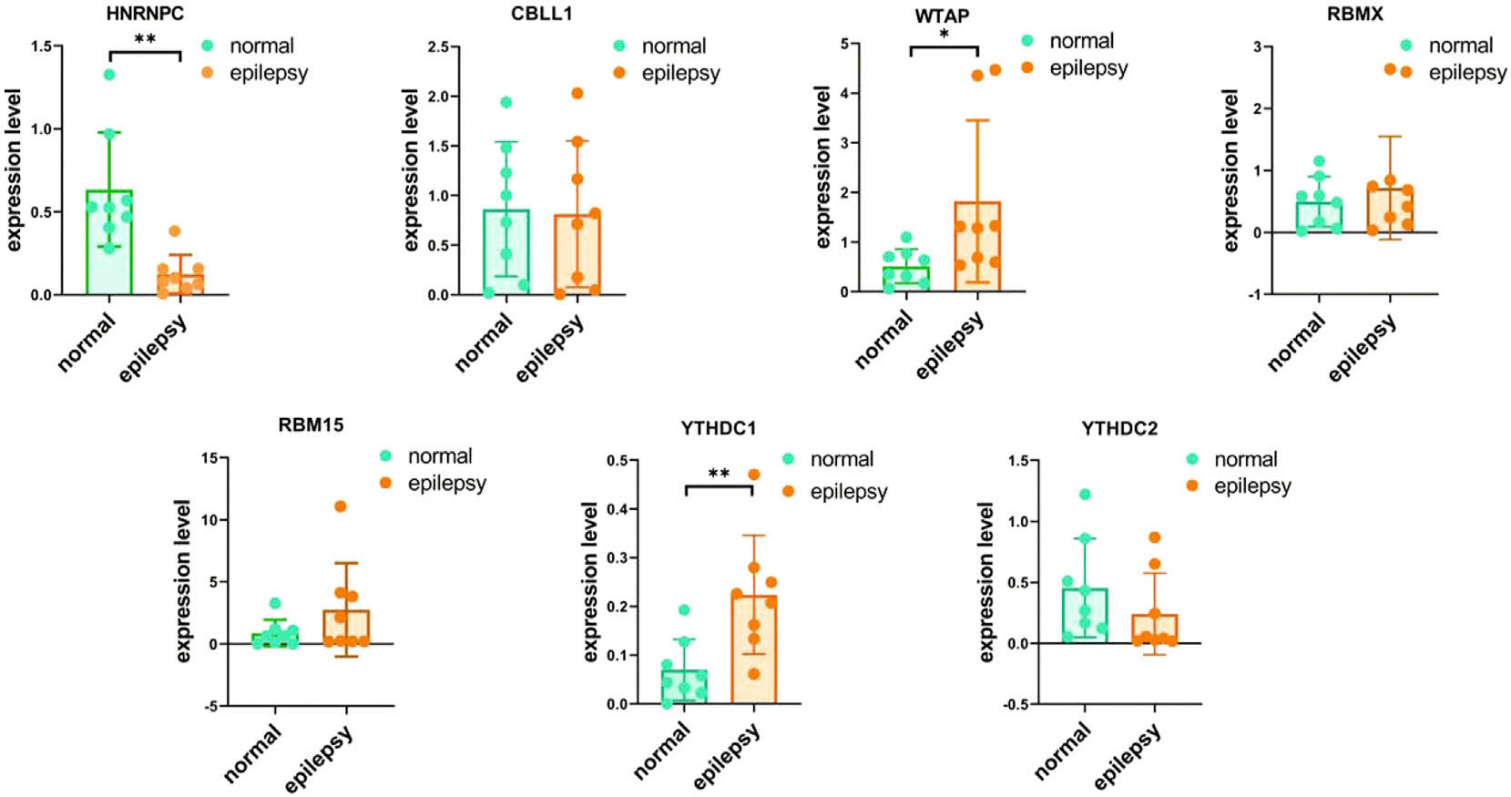
IRNA expression of seven M6A-related genes were measured in primary epilepsy and healthy samples. RNA expression of HNRNPC, CBLL1, WTAP, RBMX, RBM15, YTHDC2, and YTHDC1 were measured in blood samples using qRT-PCR. *p*-values were calculated using a two-sided unpaired Student’s *t*-test. *13 < 0.05 **p < 0.01.

To validate the significant m6A regulator expression levels, qRT-PCR was used to check the 16 clinical blood samples from eight patients with epilepsy and eight epilepsy people. The qRT-PCR results indicated that WTAP and YTHDC1 have higher expression levels in patients with epilepsy, while HNRNPC was expressed to a lower level, which is consistent with our integrated analysis (Figure 12). We also checked the expression levels of M6A related genes with the comparison of drug using (Supplementary Figure S3) and provided other writer and eraser genes’ expression condition (Supplementary Figure S2).

## Discussion

Epilepsy is a disease caused by the abnormal discharge of neurons (Manford, 2017). Recent research has shown that m6A regulators participate in the occurrence and progression of epilepsy, although their functional roles remain unclear (Zhang et al., 2022). Therefore, we sought to investigate the function of m6A regulators in epilepsy, as well as possible molecular pathways that they participate in.

Firstly, we analyzed the expression of 17 m6A-related genes extracted from a public dataset and found that seven of them (HNRNPC, WTAP, RBM15, YTHDC1, YTHDC2, CBLL1, and RBMX) were differentially expressed between patients with epilepsy and healthy individuals. We then ranked the genes by their importance through establishing an RF model. A nomogram based on the seven genes was established to predict the occurrence of epilepsy and was verified to be beneficial to patients with epilepsy through the DCA curve. However, due to the lack of genes in this data, only 17 m6A-related genes were extracted. Heterogeneous nuclear ribonucleoprotein C (HNRNPC), a nuclear RNA-binding protein involved in pre-RNA processing (Liu et al., 2015), has been shown to play an important role in alternative splicing, cell cycle, and the invasion of cancer cells (Hu et al., 2021). Moreover, Wilms tumor 1 associated protein (WTAP), is a conserved protein in the cell nucleus as the partner of Wilms tumor 1 (WT1) (Little et al., 2000), and has been found to correlate with alternative splicing, cell cycle regulation, and X-chromosome inactivation (Chen et al., 2019). RNA binding motif protein 15 (RBM15), whose function is to bind the m6A complex and recruit it to a unique RNA site, also takes part in numerous regulations, including the proliferation and migration of tumor cells, and macrophage infiltration (Zeng et al., 2022). YTH domain containing 1 (YTHDC1), a gene that can bind to m6A-modified RNAs and facilitate the selection of splice site (Xiao et al., 2016), and YTH domain containing 2 (YTHDC2), which is involved in the regulation of mRNA decay (Lin et al., 2017), are members of the YTH family, and play a crucial role in the phase-shift of mitosis and meiosis with different binding affinities of m6A (He et al., 2021). Cbl Proto-Oncogene Like 1 (CBLL1), an E3 ubiquitin ligase with a RING-finger domain (Huang et al., 2020), was found to have an upper expression in non-small-cell lung cancer tissues and was identified to be involved in the cell cycle and colony formation. RNA binding motif protein, X-linked (RBMX), also known as hnRNPG, has recently been rediscovered as participating in DNA damage repair (Zheng et al., 2020), while the absence of this gene can lead to aberrant activation of the p53 pathway (Cai et al., 2021). According to numerous studies, we found that the seven genes have an apparent relationship with the cell cycle, as well as the migration and progression of tumor cells. Unfortunately, few reports have evaluated the relationship between these seven genes and epilepsy. It is hoped that this research will provide a new direction for further research on m6A regulators and epilepsy.

Certain findings have presented the close relationship between epilepsy and the immune system (Perucca et al., 2020). Some scholars have proposed “autoimmune epilepsy” as a systematic explanation (Husari and Dubey, 2019). Autoantibodies against neuronal surface antigens play a substantial role in target proteins (Geis et al., 2019), resulting in the excitement and damage of synaptic function and plasticity. Simultaneously, nerve-specific antibodies commonly associated with autoimmune epilepsy include leucine-rich glioma inactivation protein (LGI1), glutamic acid decarboxylase (GAD), and glutamate decarboxylase 65 (GAD65) IgG (Karaaslan et al., 2017; Nóbrega-Jr et al., 2018). According to our study, the differential expression of m6A is closely related to the component differences in immune cells, which also provides a basis for future studies of the relationship between m6A and autoimmune epilepsy.

Neuron loss and degeneration are characteristic pathological changes of epilepsy (Farrell et al., 2017; Becker, 2018), which have been verified in animal models (Li et al., 2021); however, whether these characteristic or non-characteristic neuronal changes and death are associated with programmed cell death remains unknown. Here, we analyzed the correlation of several unique cell death modes, such as cuproptosis, pyroptosis, and ferroptosis, and concluded that in the study of differences caused by m6A and gene grouping there is a strong correlation between iron death and epilepsy. Finally, diabetes has also been identified as a high-risk factor for epilepsy (Marcovecchio et al., 2015; Shlobin and Sander, 2020). Therefore, we also conducted an analysis on metabolism. The enrichment results and grouping studies confirmed a close relationship between the TAC, pyruvate metabolism, and type II diabetes, indicating that cluster B may be resistant to insulin and glycometabolism disorder. Frye et al., 2018.

## Conclusion

In this study, seven candidate m6A regulators were selected and a nomogram model was established to predict the prevalence of epilepsy. We identified two m6A patterns and found one may be related to immune epilepsy. A clinical correlation heatmap was drawn and found a unique association between m6A gene clustering and drug response. Through enrichment analysis, the exact association between m6A gene clustering and ferroptosis and glucose metabolism was preliminarily found. By analyzing pathways of glucose metabolism, we found that cluster B may be related to type II diabetes. Taken together, these results show that m6A-related genes may regulate the immunologic process, cell death, drug-response, and glucose metabolism in patients with epilepsy. The detection of RNA content also confirmed the difference in these m6A genes between patients with epilepsy and healthy individuals.

## Data Availability

The original contributions presented in the study are included in the article/Supplementary Material, further inquiries can be directed to the corresponding authors.
