# Supplementary material for: Identification of RNA N6-methyladenosine regulation in epilepsy: Significance of the cell death mode, glycometabolism, and drug reactivity
Source: Front Genet. 2022 Nov 17;13:1042543. doi: 10.3389/fgene.2022.1042543 (PMC9714553; doi:10.3389/fgene.2022.1042543)
Supplement: Supplementary file 2 [file Table1.DOCX]

Homo-HNRNPC-142F GCAACGTTACCAACTTGACAGA

Homo-HNRNPC-142R TTATGAACAGAGCAGCCCACA

Homo-WTAP-161F GCAACAACAGCAGGAGTCTG

Homo-WTAP-161R TCGCTGGGTCTACCATTGTT

Homo-RBM15-197F AGTAGACTTTGCCTACACCG

Homo-RBM15-197R GTGGCACCCAATCAGAGTCA

Homo-YTHDC1-182F AGGAAAGTCAGCCACAGAGT

Homo-YTHDC1-182R GCGTAGGAGATTTGGCCCTC

Homo-YTHDC2-124F GGCCTCTCAAAAACGTGCAG

Homo-YTHDC2-124R CTCCCACCCATCACTTCGTG

Homo-CBLL2-76F TGGGTGGTCTTGATGTTCGC

Homo-CBLL2-76R GGTGCAGGTTTCGCTTTGTT

Homo-RBMX-126F AACCGCTGCCCTCTCGTAG

Homo-RBMX-126R GTGGTGGTGGTGCATAATCTC
