# Supplementary figures and images for: Identification of RNA N6-methyladenosine regulation in epilepsy: Significance of the cell death mode, glycometabolism, and drug reactivity
Source: Front Genet. 2022 Nov 17;13:1042543. doi: 10.3389/fgene.2022.1042543 (PMC9714553; doi:10.3389/fgene.2022.1042543)

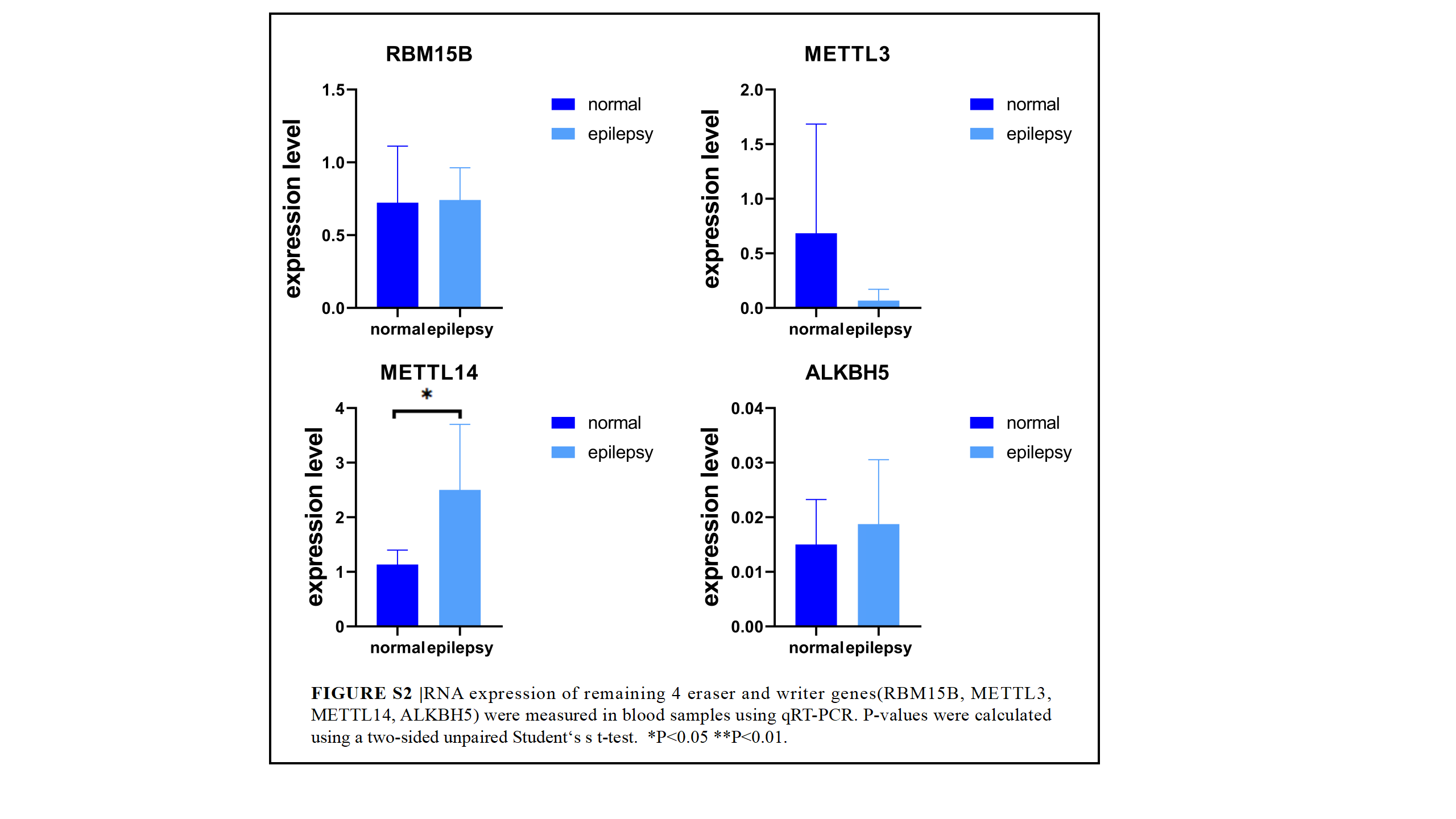

Supplement: Supplementary file 3 [file Image3.TIF]

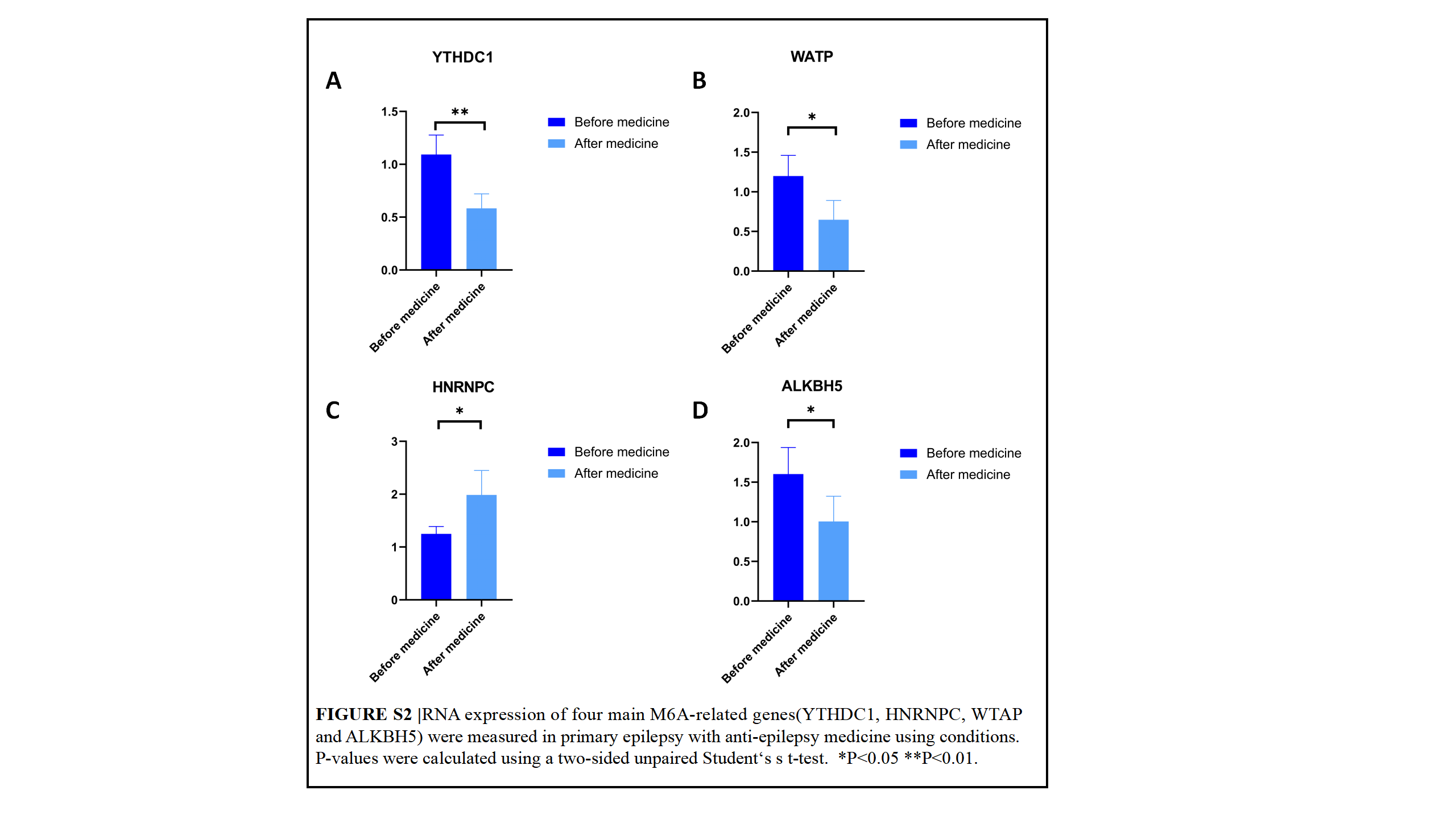

Supplement: Supplementary file 4 [file Image4.TIF]

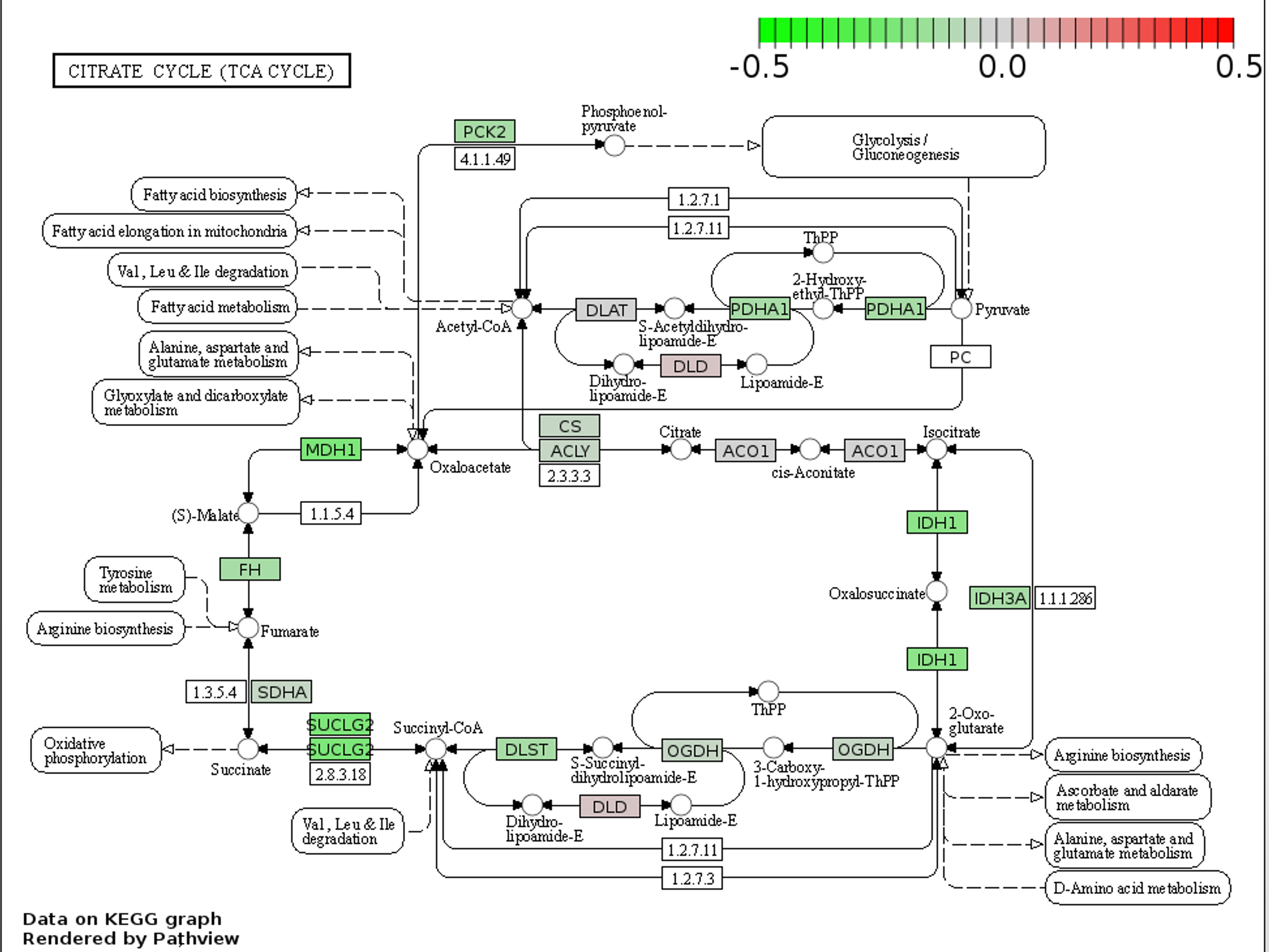

Supplement: Supplementary file 5 [file Image2.TIF]

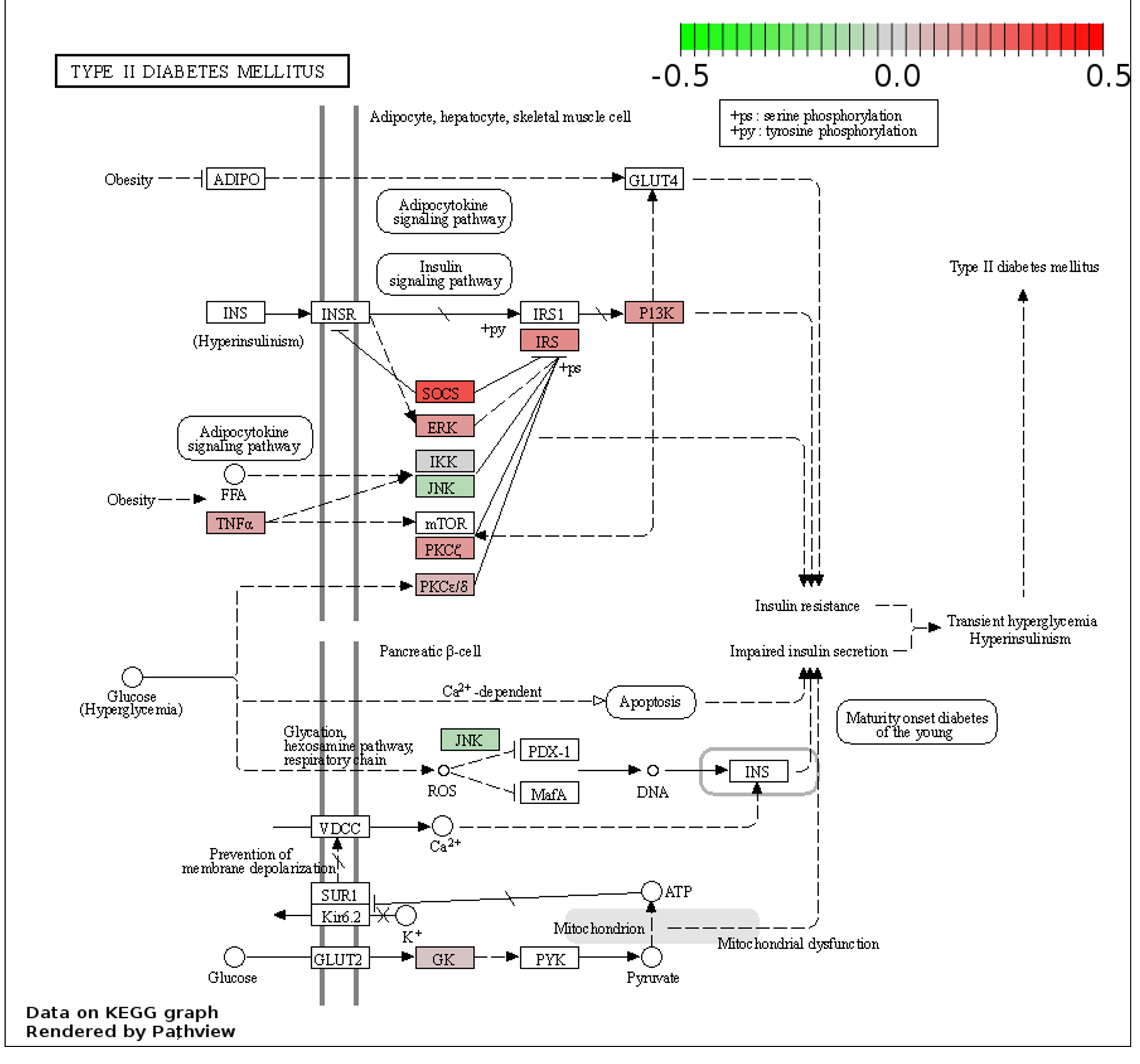

Supplement: Supplementary file 6 [file Image1.TIF]
